# Unusual Presentation of an Inguinal Bladder Hernia

**DOI:** 10.7759/cureus.35594

**Published:** 2023-02-28

**Authors:** Bradley Casey, Reese Hofstrand, Amol Bahekar, Divyang Patel, Bhaskar Chhetri

**Affiliations:** 1 Internal Medicine, Cape Fear Valley Medical Center, Fayetteville, USA; 2 Cardiology, Cape Fear Valley Medical Center, Fayetteville, USA

**Keywords:** urinary urgency, general surgery consult, urology consults, chronic kidney disease (ckd), bladder hernia

## Abstract

Cases of an inguinal bladder hernia (IBH) are rare as the diagnosis may be challenging because patients are often asymptomatic or have nonspecific symptoms. When patients are symptomatic, normally they complain of urinary symptoms. Our patient initially presented to the hospital because he had a ground-level fall after having chest pain while transitioning from a bed to a wheelchair. Incidentally in the emergency department, he was found to have scrotal edema, which was later diagnosed as inguinal bladder herniation. The patient did not have any further episodes of chest pain or abdominal pain once he was given medicinal therapy for his IBH. Surgery is usually the definitive treatment for inguinal bladder herniation, but our patient wished to try medicinal therapy and follow-up outpatient.

## Introduction

Inguinal bladder hernias (IBHs) are a rare disorder, and it has been reported that IBHs account for 0.4-3% of adult inguinal hernia cases in western countries [1]. Although a bladder hernia frequently goes undetected, it can occasionally cause vague symptoms like lower urinary tract discomfort or urinary tract infections [2]. Patients with an IBH run the risk of being misdiagnosed due to the disease's rarity [3]. Here, we present an interesting case of a patient who has been having intermittent chest and abdominal pain and endorses that his abdominal and chest pain started as his scrotum increased in size. The patient was later diagnosed with a large IBH by imaging. 

## Case presentation

**Figure 1:**
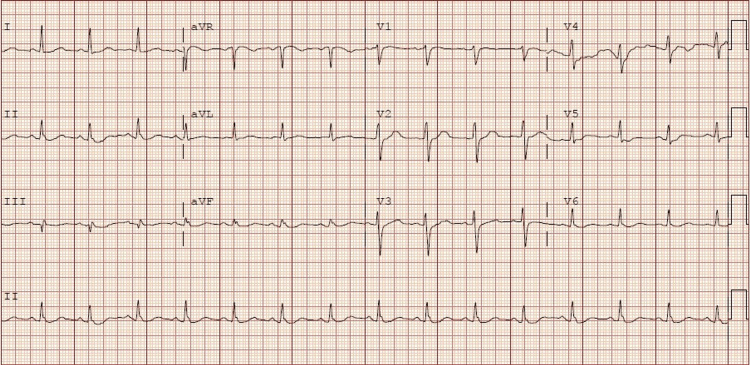
Sinus rhythm 90 beats per minute and no significant ST or T wave abnormalities

An 84-year-old male with a past medical history of right nephrectomy secondary to renal mass and chronic kidney disease stage IV arrived at the emergency department after a ground-level fall at home while transitioning from a living room chair to a wheelchair. The family was unable to lift him off the ground, so they called emergency medical services (EMS) for assistance. When EMS arrived, he had been on the floor for approximately 70 minutes, and they recommended taking the patient to the emergency department for evaluation due to the patient complaining of intermittent chest pain. The patient reported that over the past two months, he had noticed that his scrotum had increased in size. While his scrotum was increasing in size, he noticed that he was developing suprapubic abdominal pain that was radiating to his inferior sternum. He was unable to fully describe his chest and abdominal pain but endorsed that it was worse when he was trying to strain to urinate. Initial vital signs were blood pressure of 103/47 mm/hg, pulse 95 beats per minute, temperature 98 °Fahrenheit, respiratory rate 28 breaths/min, and oxygen saturation on room air 99%. Initial labs are available in Table 1. Due to patient's complaint of chest pain, he had an electrocardiogram (Figure 1), which showed a sinus rhythm of 90 beats per minute and no significant ST or T wave abnormalities. The patent was started on aggressive intravenous hydration due to rhabdomyolysis. 

**Table 1:** Patient's initial blood work when he arrived at the emergency department

Initial Laboratory Tests	Normal Range	Patient's Lab Test
Complete Blood Count		
White Blood Cell Count	4.5 - 12.5 x10*3/uL	18.0 x10*3/uL
Hemoglobin	13.5 - 18.0 g/dL	10.0 g/dL
Mean Corpuscular Volume	81.0 - 99.0 fL	93.2 fL
Platelets	150 - 450 x10*3/uL	219x10*3/uL
Comprehensive Metabolic Panel		
Sodium	136 - 145 mmol/L	140 mmol/L
Potassium	3.5 - 5.1 mmol/L	5.0 mmol/L
Blood Urea Nitrogen	7 -18 mg/dL	75 mg/dL
Creatinine	0.55 - 1.30 mg/dL	4.94 mg/dL
Glucose	74 - 106 mg/dL	144 mg/dL
Aspartate Aminotransferase	15 - 37 U/L	23 U/L
Alanine Transaminase	12 - 78 U/L	53 U/L
Glomerular Filtration Rate	>60.0 mL/min/1.73m*2	16 mL/min/1.73m*2
Urinalysis		
Urine Specific Gravity	1.010 - 1.025	1.011
Urine pH	5.0 - 8.0 pH	5
Leukocytes Urine	Negative	Negative
Nitrites urine	Negative	Negative
Protein Urine	Negative	Negative
Glucose Urine	Negative	Negative
Bilirubin Urine	Negative	Negative
Blood Urine	Negative	Negative
Red Blood Cell Urine	0-3 High Powered Field	5 High Powered Field
Other Laboratory Testing		
Creatinine Kinase	39 - 308 U/L	3,922 U/L
High-Sensitivity Troponin	0 -79 ng/L	40 ng/L
Procalcitonin	0.07 - 0.50 ng/mL	1.34 ng/mL

**Figure 2:**
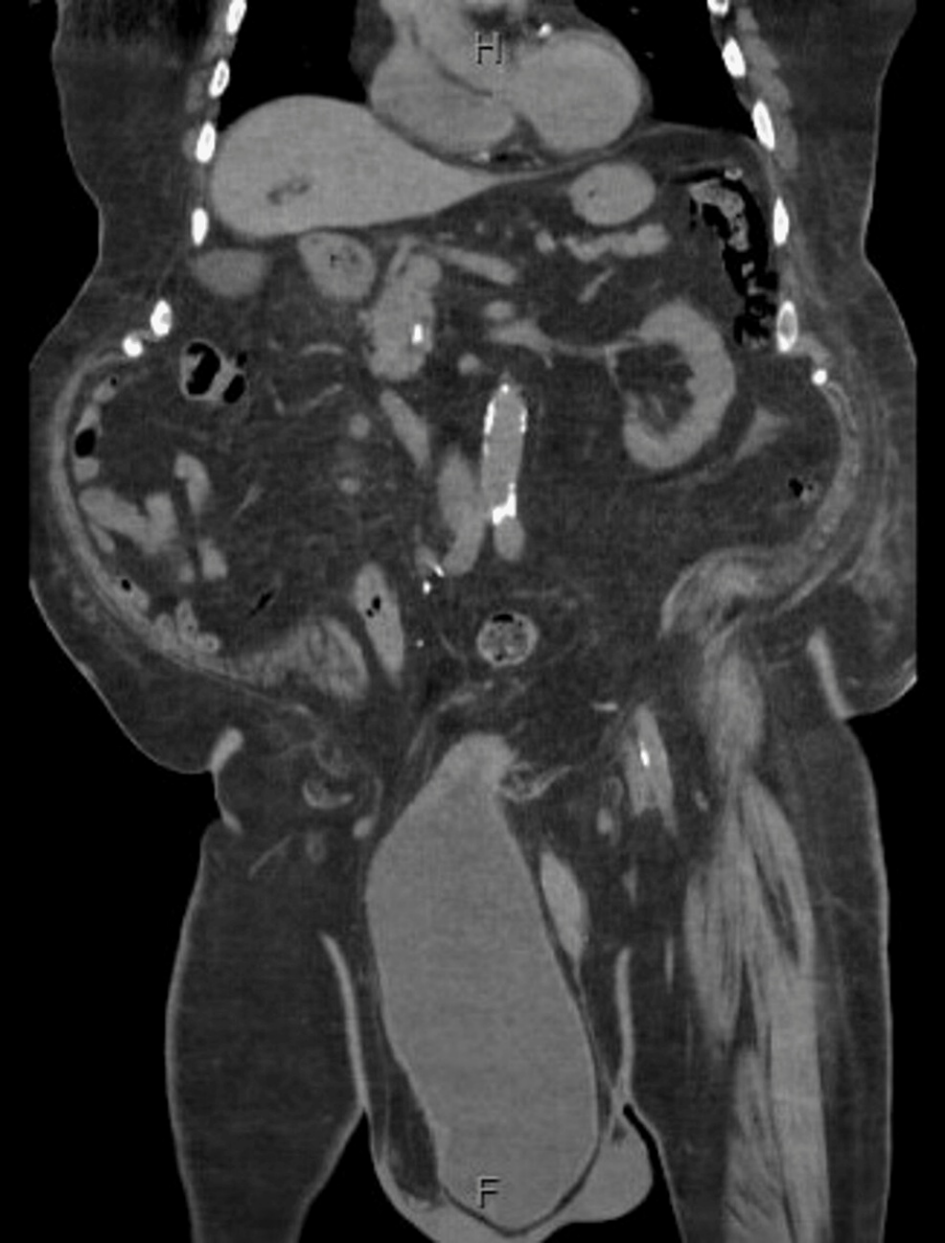
CT scan of abdomen and pelvis coronal view showing a large right inguinal hernia containing most of the bladder and the appendix CT: Computed tomography

**Figure 3:**
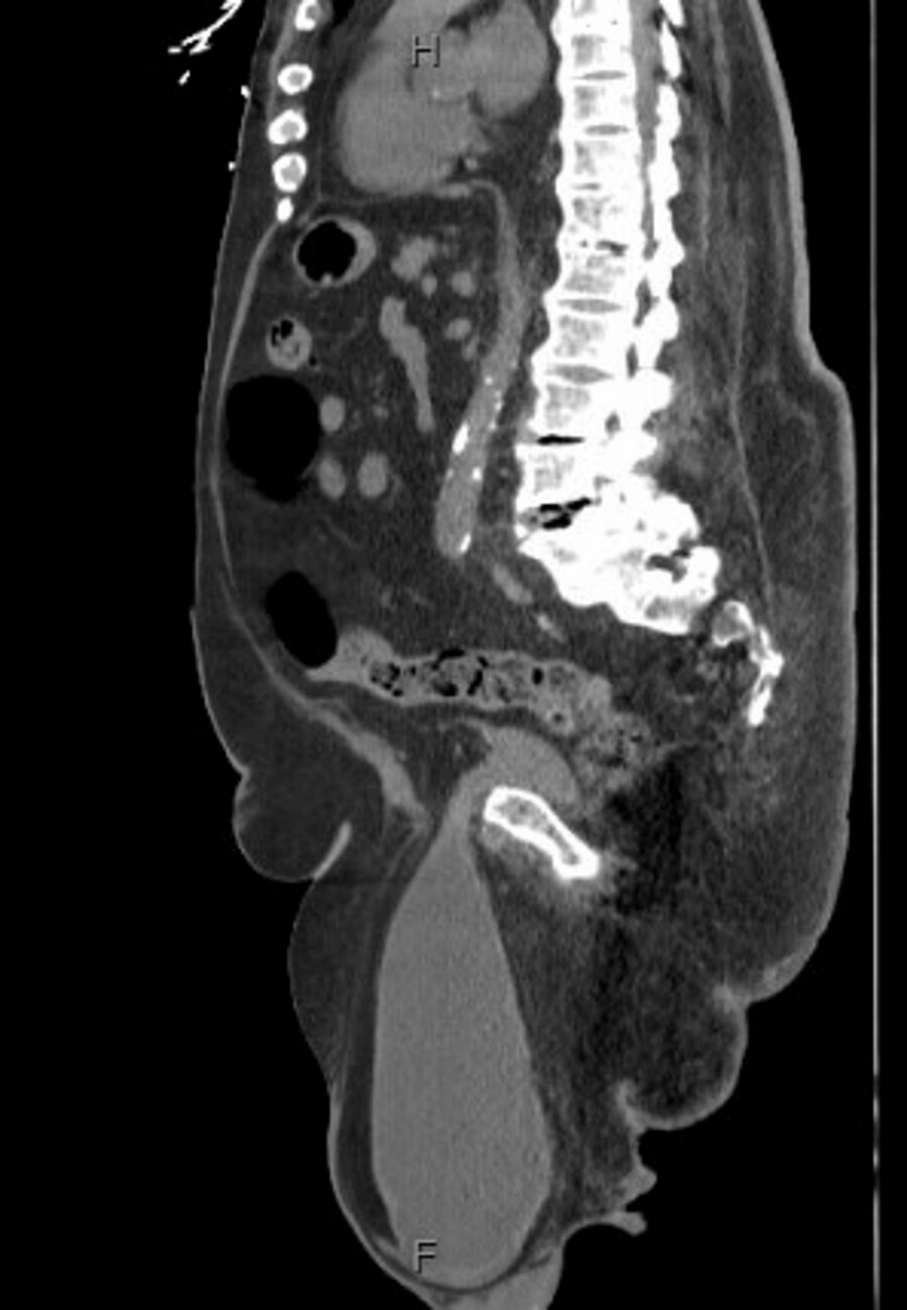
CT scan of abdomen and pelvis sagittal view showing a large right inguinal hernia containing most of the bladder and the appendix CT: Computed tomography

During the physical examination that was performed in the emergency department, he was seen to have a large non-tender scrotum. An ultrasound was ordered to better assess his scrotum. The ultrasound showed that he had a very large fluid collection in the scrotal sac, and they recommended computed tomography (CT) scan to better evaluate. CT scan (Figures 2, 3) showed large right inguinal hernia containing most of the bladder and the appendix. Urology and general surgery service were consulted for evaluation, and they both recommended the patient to undergo surgery for his large inguinal bladder herniation. Urology attempted to reduce the cystocele containing the bladder manually, but the patient refused secondary to pain. At this time, the patient said that he wanted to wait until the rest of his family arrived at the hospital before he made any decisions about surgery. CK improved with intravenous fluids, but the patient was noted to have ongoing urinary retention. A bladder scan at that time showed 650 cc of urine; the patient refused straight catheterization or indwelling catheterization and received tamsulosin for ongoing retention. The patient eventually declined any surgical intervention and reported that all of his symptoms including chest and abdominal pain had resolved. He was given the option to go home with an indwelling foley catheter, but he wanted to continue with tamsulosin only at this time. Before being discharged, the patient was urinating without difficulty, and his chest pain had completely resolved by the time of discharge. The patient was eventually discharged from the hospital and was lost to follow-up. 

## Discussion

Inguinal hernias are thought to have both a congenital and an acquired component, and some data point to the possibility that genetics may also be involved [4]. Patients with an inguinal hernia are at least four times more likely to have one than those without a known family history. Ninety percent of all inguinal hernias are in men, compared to 10% in women with a bimodal distribution of incidence peaks around age 5 and after age 70. Indirect hernias are the most typical groin hernia in both males and females, accounting for two-thirds of all hernias. The indirect hernia sac is formed inside the spermatic fascia from the peritoneum passing through the deep inguinal adjacent to the vas deferens and pampiniform plexus lateral to the inferior epigastric vessels [5].

A small inguinal bladder herniation usually presents with no symptoms, but a large IBH tends to produce symptoms including swelling in the groin and/or symptoms of lower urinary tract infection [6]. Patients with inguinal bladder herniations may also present with dual voiding, depending on the size of the herniation. A dual voiding occurs in patients with an IBH when the bladder empties naturally followed by manual compression of the hernia to facilitate complete voiding [6]. A voiding cystourethrogram is one of the best diagnostic imaging methods for bladder herniation, and this method may reveal a dog-ear-shaped bladder [7]. Ultrasonography in IBHs is mainly used to assess the complete urinary system, especially in older patients to assess for a blocked bladder outlet and a clinical suspicion of bladder hernia. Ultrasonography may show a hypoechoic mass lesion emanating from the bladder through the inguinal canal or the scrotum if bladder outlet tract obstruction is the cause. While a CT scan is not the first-line imaging study, it can be used to help when other studies are indeterminate [1,2].

IBHs are typically treated surgically with a herniorrhaphy [8]. When there is evidence of a bladder tumor, herniated bladder diverticulum, a tight hernia neck, or bladder wall necrosis, resection is the current treatment modality. A mesh can be used to repair the hernia in order to prevent a recurrence, but some patients may prefer to follow a conservative treatment plan that involves watchful waiting or intermittent self-catheterization [8]. Our patient chose the conservative pathway with waiting and without intermittent catheterization. He chose to follow up outpatient for evaluation, and prior to discharge, he had complete resolution of his abdominal and chest pain with only tamsulosin. Tamsulosin is a selective alpha-1 blocker and has been FDA-approved to treat benign prostatic hyperplasia [9]. It is believed that alpha-1 blockers tend to relax smooth muscle in the prostate, which may allow urine to flow more freely through the urethra. 

We would expect abdominal pain in certain patients with a large IBH, but chest pain has not been documented with IBHs. During our literature review, we were not able to find any cases that showed patients with a large IBH causing associated chest pain. 

## Conclusions

Here, we presented an interesting case of a patient who had been complaining of intermittent chest and abdominal pain that was caused by his large IBH. He was diagnosed with an IBH and chose to not pursue surgical intervention but only a medicinal approach. With medicinal therapy, his scrotal swelling improved, and eventually his chest pain completely resolved. Normally patients with IBHs experience urinary symptoms, but from a literature review, chest pain has never been associated. This case also brings an important focus on physical examination, as our patient did not initially present with the complaint of scrotal edema. Without a thorough physical examination revealing scrotal edema, his initial differential with abdominal and chest pain would have been vast. 
